# Diagnosis and Prognosis of Retroperitoneal Liposarcoma: A Single Asian Center Cohort of 57 Cases

**DOI:** 10.1155/2021/7594027

**Published:** 2021-04-01

**Authors:** Jianchun Xiao, Jianghao Liu, Minting Chen, Wei Liu, Xiaodong He

**Affiliations:** ^1^Department of General Surgery, Peking Union Medical College Hospital, Chinese Academy of Medical Sciences & Peking Union Medical College, No. 1 Shuaifuyuan, Beijing 100730, China; ^2^Chinese Academy of Medical Sciences & Peking Union Medical College, Beijing 100005, China

## Abstract

**Background:**

Liposarcoma is a soft tissue malignancy, commonly observed in the extremities. However, retroperitoneal liposarcoma is seldom reported and its diagnosis is frequently neglected. This study aims to present the clinical characteristics, diagnosis, and prognosis of five liposarcoma subtypes and report our experience of patient treatment.

**Methods:**

We conducted a single-center noninterventional retrospective study of 57 retroperitoneal liposarcoma patients admitted to Peking Union Medical College Hospital (PUMCH, Beijing, China) between July 2011 and December 2019. We collected and analyzed their demographic, clinical, imaging, histological, therapeutic, and prognostic data over a mean 4.5-year follow-up period.

**Results:**

Twenty-five (44%) patients were asymptomatic prior to diagnosis, with abdominal distension as the chief complaint in 18 (32%) patients and abdominal pain observed in 16 (28%) patients. Masses were evaluated by computed tomography (*n* = 48, 84%) or ultrasound (*n* = 25, 44%). Laparotomy (*n* = 52, 91%) was the dominant therapeutic modality rather than laparoscopy (*n* = 5, 9%). All patients were treated with R0 resection except two patients who underwent R2 resection. We conducted regular follow-ups every six months after surgery for a mean duration of 4.5 years. Recurrence was experienced by 14 (25%) patients and a further 9 (16%) died during follow-up.

**Conclusions:**

Abdominal distension and pain are chief complaints with liposarcoma. As the extremities are the main liposarcomas locations, the diagnosis of retroperitoneal liposarcoma is usually neglected. Since half of the patients are asymptomatic, timely diagnosis and treatment are highly dependent on regular ultrasound and computed tomography imaging. R0 resection is the key to retroperitoneal liposarcoma treatment. In comparison, patients who underwent R2 resection, which is considered a palliative treatment, had bad prognoses. Large, symptomatic dedifferentiated, and pleomorphic liposarcomas are more likely to have poor prognoses, while the prognosis for well-differentiated or myxoid liposarcoma is relatively good.

## 1. Introduction

Accounting for only approximately 10% of all soft tissue sarcomas and 15% of all sarcomas, liposarcoma (LPS) is a frequently observed tumor derived from adipocytic differentiated primitive mesenchymal cells. Its incidence peaks in the age range 50–60 years. [1, 2]. Although it occurs predominantly in the deep soft tissues of the extremities, LPS is also reported in the abdomen in areas such as the esophagus, stomach, and descending mesocolon [3, 4]. The retroperitoneum is a rare LPS location, with only a few publications discussing its diagnosis, clinical characteristics, and prognosis [2, 5, 6]. LPS is generally classified into five subtypes: well-differentiated LPS (WDLPS), dedifferentiated LPS (DLPS), myxoid LPS (MLPS), pleomorphic LPS (PLPS), and mixed LPS. WDLPS is the most common retroperitoneal LPS, accounting for 40–45% of all LPS [2, 7–9]. Gene amplification in the 12q12-21 and 10p11-14 chromosomal regions is often observed in WDLPS and DLPS, and DLPS is also associated with at 6q23 and 1p32 [8, 10]. In addition, there is an approximately 10% probability of WDLPS converting to DLPS, a more invasive LPS subtype [8]. Approximately 95% of MLPS patients have a t(12;16) (q13;p11) reciprocal chromosomal translocation resulting in an in-frame fusion of the RNA-binding protein fused in sarcoma (FUS) gene to the DNA damage inducible transcript 3 (DDIT3) gene while the remaining 5% of patients exhibit a t(12;22) (q13;q12) translocation [8, 11]. To date, no consistent chromosomal or molecular aberration has been reported for PLPS, the rarest of the subtypes [8]. Diagnosis of LPS is currently highly dependent on pathological findings, with computed tomography (CT) or magnetic resonance imaging (MRI) responsible for the majority of presurgical diagnoses [11, 12]. Since many liposarcomas can be asymptotic before diagnosis, and symptoms, if there are any, are mainly nonspecific such as abdominal pain or distension, the preoperative diagnosis of LPS is difficult [2, 13]. Although the gold standard for diagnosis remains biopsy, imaging is currently a widely accepted diagnostic tool [2, 14]. The presence of macroscopic fat on imaging suggests the presence of LPS.

Upon diagnosis, surgery is the primary recommended treatment for retroperitoneal LPS; however, the extent of resection remains controversial [15]. Although it is conventional to only resect directly involved adjacent organs, a more aggressive approach, which proposes partial resection of adjacent uninvolved organs, has also been suggested [15, 16]. Several phase II or III clinical trials have found that chemotherapy, such as trabectedin and eribulin, may improve LPS prognosis [17, 18]. Immunotherapy for LPS is now also under development.

The prognosis of LPS is highly dependent on the surgical approach and the histological subtype. WDLPS, together with low-grade MLPS, has a 5-year survival rate above 90%. In contrast, the 5-year survival rates of PLPS, DLPS, and high-grade MLPS are all below 75%, with PLPS showing the lowest of only 50% [2, 19].

## 2. Methods

The medical records of all retroperitoneal LPS patients presenting at Peking Union Medical College Hospital (PUMCH), Beijing, China, between July 2011 and December 2019 were retrospectively reviewed. Informed consent was obtained from each patient. The study was approved by the Institutional Review Board of PUMCH.

Detailed demographic and clinical data, such as histological subtype of tumor, symptoms, and physical signs, from all 57 patients were reviewed. All available imaging examinations, including ultrasound, computed tomography (CT), and MRI, were also collected. In addition, the surgical approach, together with surgical details such as surgery duration and pathological findings, was recorded. To assess the prognosis, the patients were interviewed regularly to obtain details of any relapse, postsurgical chemotherapy, or other adjuvant therapies. Regular follow-ups every six months after surgery, including CT imaging and tumor marker measurements, were conducted for all patients, with the most recent follow-up in February 2020. All data were recorded and analyzed using Python 3.7. Descriptive data were expressed in numbers (%) for categorical variables and means (SD) for continuous variables, as appropriate. Categorical variables were assessed using Pearson *χ*^2^-test or Fisher's exact test where appropriate. A *t*-test was used to analyze continuous variables. The overall survival and disease-free survival were correlated to symptoms, LPS pathological type, tumor diameter, and organ invasion using the Kaplan–Meier method. All tests were two-sided with *P* < 0.05 considered statistically significant.

## 3. Results

A total of 57 patients (26 males and 31 females) suffering from retroperitoneal LPS were recruited from PUMCH. The mean age at treatment was 57.0 (12.2) years. These patients were followed up regularly with the mean duration of follow-up being 4.5 (2.6) years.

Collation of the history of the current illness demonstrated that 25 (44%) patients had been completely asymptomatic, LPS having been detected during routine medical examinations. Abdominal discomfort was the most common symptom, with 18 (32%) patients complaining of abdominal distension and 16 (28%) complaining of abdominal pain, including 2 (4%) who had complained of both. Besides abdominal discomfort, lower extremity symptoms, including swelling and pain, were also reported in 6 (11%) patients. Unsurprisingly, larger tumor size was directly related to complaints of discomfort. For those whose tumor was less than 15 cm, about half (46%) experienced no abdominal discomfort, whereas only 33% of patients with a tumor larger than 25 cm had no abdominal discomfort. Mixed LPS tumors were the largest with an average size of 23.1 cm while MLPS tumors showed the smallest average size of only 13.1 cm. However, considering the size of our cohort, no solid conclusion can be reached. Besides, the chief complaints differ among the five LPS subtypes. Half of the WDLPS patients and 75% of the PLPS patients were asymptomatic before diagnosis while 57% of MLPS patients experienced abdominal distension as their chief complaint, and 48% of DLPS patients complained of abdominal pain (Table 1).

Detailed physical examination (PE) was conducted on each patient. Only 18 (32%) among the total of 57 had no apparent positive signs of the disease, the retroperitoneal mass tangible in all the other 39 (68%) patients. Among the 39 patients with tangible mass, 29 (74%) had little mobility, and 29 (74%) had a clear margin. Tenderness pain was reported by only 14 (36%) patients. As for subtypes, half of WDLPS patients had tangible retroperitoneal masses while the other half did not. Among 23 DLPS patients, retroperitoneal masses of 16 (70%) patients were tangible, and 7 (30%) were not tangible. All PLPS and mixed LPS patients had tangible retroperitoneal masses (Table 1).

**Figure 1:**
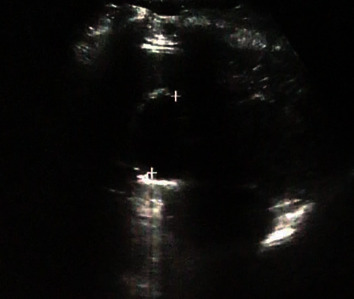
Ultrasound of retroperitoneal MLS patient. Retroperitoneal MLS, indicated by while “+” symbols.

**Figure 2:**
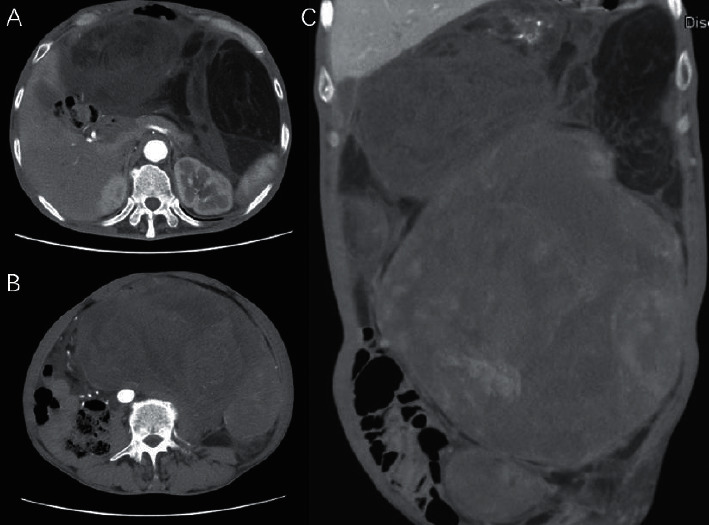
CT scan of retroperitoneal MLS patients. (a) Huge retroperitoneal MLS visible at kidney level. (b) Huge retroperitoneal MLS visible at colon level. (c) Huge retroperitoneal MLS, coronal view.

For all 57 patients, retroperitoneal LPS was diagnosed via presurgical imaging and postsurgical pathological analysis. The form of presurgical imaging used for diagnosis included ultrasound (Figure 1), CT (Figure 2), and MRI. All cases of LPS were located within the retroperitoneum and in 7 (12%) patients the tumor mass had also invaded the pelvis. In 53 (93%) patients, the tumors were situated in only half of the retroperitoneum, 29 of which were on the left side and 24 on the right side. No family history of LPS was reported in any of the 57 patients (Table 1). Patients were divided into three subgroups according to maximum tumor diameter indicated by imaging examination (larger than 25 cm, medium, or smaller than 15 cm) (Table 2). Certain characteristics observed on ultrasound or CT, such as echoic and density, were not found to be predictive presurgical factors for pathological subtypes: both mixed and low CT densities were reported in each subtype, and no significant differences were observed in the ratios (Table 3).

All 57 patients received surgery, of which 52 (91%) surgeries were open and 5 (9%) were laparoscopic; all laparoscopic surgeries were performed because of a tumor size less than 10 cm. Besides tumor tissue resection, the invaded organs were also removed during surgery while all nearby uninvolved organs were retained. The most commonly involved organs were the pancreas and kidney. Five (9%) patients had their pancreas resected; of these, four were pancreas resection, including head, body, and tail, and one had only the head resected. Two (4%) patients had one-side kidney removals. In 10 patients, the LPS tumor had grown to surround the kidney without direct involvement of the kidney; these tumors were completely resected since a macroscopic fissure existed between the tumor and the kidney. All postsurgical histopathological reports were obtained for analysis; the diagnosis of LPS was confirmed in every patient. Bleeding during surgery ranged from 30 to 8400 mL, with a mean of 910 mL. The 8400 mL bleeding occurred during separation of the tumor mass from the psoas major, and, unfortunately, we lost contact with this patient two years after surgery. The surgery duration ranged from 2 to 8 hours with a mean of 4.18 hours. Neither surgical bleeding nor duration was related to the size of LPS mass (*R*^2^ for bleeding and size: 0.02, *R*^2^ for duration and size: 0.03). Fourteen (25%) patients were admitted to the intensive care unit (ICU) in PUMCH for better postsurgical care.

The mean duration of hospital stay was 18.6 (8.9) days (7.6 days prior to surgery and 11.0 days after surgery). For most patients, diagnosis and differential diagnoses such as paraganglioma were the main reasons for the long presurgical hospital stay. In addition, it took some time to fully evaluate the adhesions between the tumor and its neighboring organs, and to further decide on the surgical modality. No significant association was observed between the length of the hospital stay and the size of the LPS (largest diameter greater than 15 cm by ultrasound or CT) (large vs. small: 17.97 vs. 19.61, *P*=0.492). Postsurgical pathological results confirmed that all patients had LPS, as well as confirming the subtype. During the average 4.5-year follow-up, 14 (25%) recurrences and 9 (18%) deaths were reported among the 57 patients. Two (4%) patients received radiotherapy, and 2 (4%) received chemotherapy following surgery. A complaint of hypoleukemia, lymphopenia, and herpes zoster was received from an 81-year-old female who underwent radiotherapy following surgery. No severe postsurgical complications were reported from other patients, such as hemorrhage or postsurgical infection.

**Figure 3:**
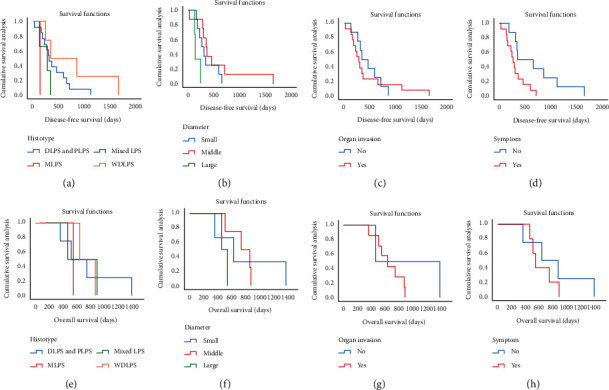
The Kaplan–Meier estimates of DFS and OS of patients with retroperitoneal LPS under different conditions. LPS: liposarcoma; DFS: disease-free survival; OS: overall survival; DLPS: dedifferentiated liposarcoma; PLPS: pleomorphic liposarcoma; MLPS: myxoid liposarcoma; WDLPS: well-differentiated liposarcoma.

Four patients received postsurgical radiotherapy or chemotherapy, the latter being standard MAID therapy comprising mesna, adriamycin, ifosfamide, and dacarbazine. Recurrence was not observed in any of these four patients during follow-up. At the last follow-up in February 2020, there had been 9 (16%) deaths and 14 (25%) recurrences, and we had lost contact with 13 (22%) patients. According to the Kaplan–Meier survival, DLPS and PLPS with larger sizes and developed symptoms were prone to having lower disease-free survival, with DLPS having the highest recurrence rate (35%) and PLPS the highest death rate (25%). There were no statistically significant differences in prognosis for the invasion of different organs (Figure 3, Table 4).

## 4. Discussion

As a subtype of sarcoma, liposarcoma accounts for approximately 15% of all sarcomas, making it the most common soft tissue sarcoma [5]. LPS mostly occurs in the extremities, followed by the retroperitoneum. There have also been reports of LPS in rare locations, such as the mediastinum, larynx, or para-testicular tissue [20–22]. The high occurrence in the retroperitoneum may be attributed to metastasis of LPS from other parts of the body, especially those where fat is abundant [1, 2, 23]. Primary retroperitoneal LPS usually originates in the perirenal fat; we observed 19 (33%) perirenal LPS tumors or instances of LPS directly invading the kidney in our cohort. LPS peaks in the range of 50–60 years, and in our 57-person cohort, the mean age at diagnosis was found to be 57.0 years with 20 (35%) patients being in 50–60 age range. We consider this phenomenon to be the result of a higher tolerance in older patients and increased severity in younger patients since PUMCH gathers severe cases of many diseases in China. There was no significant sex difference in LPS occurrence, and the ratio of our cohort was 26 (46%) males to 31 (54%) females.

The different LPS subtypes have specific genetic mutations. For example, the t(12;16) (q13;p11) reciprocal translocation results in MLPS [8, 11], while gene amplifications in the 12q12-21 and 10p11-14 regions are associated with WDLPS and DLPS, and an additional amplification in either 6q23 and 1p32 is also necessary in DLPS [8, 10]. There are no reports on possible relationships between the occurrence of LPS and exterior factors such as trauma or drug usage.

LPS is usually found accidentally or on regular physical check-ups. The reported clinical symptoms are principally abdominal pain and distension, both in the present cohort and in previously published studies [9, 24–26]. Since abdominal pain and distension are nonspecific and often tolerated by patients, it is difficult to diagnose retroperitoneal LPS or differentiate LPS subtypes via clinical symptoms. We did, however, discover that symptomatic LPS was associated with lower disease-free survival.

In terms of diagnosis using presurgical imaging, CT and MRI are regarded as the most appropriate modalities. Different subtypes can be distinguished using CT and MRI. WDLPS typically contains more than 75% adipose tissue with septations thicker than 2 mm and small internal nodular areas. Using CT, such nodular areas can be found with soft tissue attenuation. Septations and nodular areas in WDLPS show hyperintense character on T2-W1 MRI, distinguishing this subtype from the other LPS types [14]. Although similar to WDLPS, DLPS can still be identified by larger non-lipomatous components containing nodular areas [27]. MLPS often shows a multilobulated, hypoechoic structure on ultrasound [28]. Moreover, MLS usually exhibits low signal intensity in T1W and intermediate signal intensity in T2W, distinguishing it from other types of tumors [12, 25]. PLPS, due to its specific components, shows little fat attenuation on CT [14]. However, hemorrhage and necrosis occur frequently in PLPS, causing heterogeneity on imaging, making diagnosis difficult [29]. Although theoretically distinguishable, no specific subtypes were diagnosed before surgery in this cohort. Because the surgery modality was the same among all subtypes, the presurgical diagnosis of “huge retroperitoneal mass” was enough for surgery. So far, the postsurgery pathological results remain the gold standard for subtype differentiation, [14] allowing predictions of prognosis and the choice of chemotherapy or radiotherapy.

The size of the LPS tumors ranged from 1.2 × 1.2 cm to 36.6 × 26.5 cm in the present cohort, with a median diameter of 14.6 cm. Using the longest diameter of 15 cm and 25 cm as thresholds, the patients were classified into three subgroups in the Kaplan–Meier survival analysis, which demonstrated that the presurgical tumor size had a statistically significant influence on prognosis (Figure 3, Table 4).

Although the extent of resection required is still debatable, surgery is still the key treatment for LPS [30]. Traditionally, a macroscopically negative margin is sufficient for treatment, regardless of pathological subtypes. In our 57-patient cohort, all patients received R0 resection with a macroscopically negative resection margin except for two R2 resections, which were both because of older age and poor physical condition. Both patients receiving R2 resections died within one year after surgery. The first R2 resection, a 75-year-old female, had a 30*∗*15 cm LPS across her diaphragm and symphysis pubis. The lower half of the LPS was resected successfully, but the upper half was closely adherent to the liver while also surrounding the kidney tightly. Considering that the patient was in poor physical condition, after obtaining the consent of the patient's daughters, the upper half of the LPS was not resected. No adjuvant therapy was applied in this patient. The patient died of LPS 17 months after the surgery. The second R2 resection, an 80-year-old female, had a 40 cm LPS pushing the duodenum and inferior vena cava to the left side of the abdomen. This patient's LPS had rich blood supply, most of which came from the liver and right kidney. Although the major part of the LPS was resected, the remaining LPS near the liver and right kidney could not be excised. This situation was explained to her relatives, who agreed to an R2 resection as alleviation. This patient accepted no adjuvant therapy and passed away 13 months after surgery.

The histological subtype is an important factor for prognosis prediction, including local recurrence, distant recurrence, and death. Previous cohort studies have demonstrated that DLPS has the highest risk for both local and distant recurrence, while WDLPS has the lowest risk [19, 31]. In this cohort, we also found that DLPS and PLPS were associated with higher recurrence and malignancy rate (Figure 3 and Table 4) while the recurrence and death rates were lowest in the WDLPS subtype, which is consistent with previous reports. All the recurrences reported in our cohort were local. However, we did not identify a relationship between tumor invasion of surrounding organs and postoperative clinical outcomes; this aspect requires further research. The poor prognosis of DLPS encouraged surgeons to explore extended surgery including the en bloc resection of adjacent organs, even though uninvolved [15, 30]. Studies have shown that extended resection lowers the risk of local recurrence but its effect on overall survival remains unclear [16, 32]. No extended resections were performed in our cohort, due to concern about poor life quality after adjacent uninvolved organ resection.

There are other approaches to improve LPS prognosis besides the extended surgery modality, such as radiotherapy and chemotherapy. Although retroperitoneal LPS is relatively radiosensitive, so are its nearby organs. An overdose of radiotherapy causes damage to surrounding radiosensitive organs, such as the liver and kidney, so the timing and type of radiotherapy used for retroperitoneal LPS matter [2]. Among all subtypes, MLPS is the most chemo-sensitive, making chemotherapy possible [12, 17, 18]. In our cohort, four patients received postsurgery adjuvant therapy, among which three were diagnosed with MLPS by postsurgery pathological testing. None of the four patients experienced recurrence or death, demonstrating the efficiency of radiotherapy and chemotherapy, but no conclusion can be drawn due to the small sample size. Also, as this is a retrospective study, no standardized postsurgical chemotherapy or radiotherapy was given to these patients. Future guidelines may recommend regular chemotherapy or radiotherapy to postsurgical LPS patients.

There are several limitations to this study. Firstly, as a retrospective study, missing data, recall bias, and errors in the initial medical records may exist. Secondly, the sample size of 57 is relatively small, and the numbers for each subtype are in some cases less than 10. Thirdly, as PUMCH is among the most comprehensive third-grade class-A hospitals in China, the high number of severe and difficult surgical cases seen may give rise to bias.

We collected and analyzed the detailed demographic and clinical data of all 57 patients. We found that presurgery imaging helped to diagnose LPS, and specific subtypes could also be distinguished via CT or MRI. The prognosis for the different subtypes differed. Recurrence and death occurred more frequently in symptomatic patients with larger DLPS and PLPS tumors; in contrast, WDLPS had relatively low recurrence and death rates. A macroscopically negative margin was the surgical goal for most of the patients in this cohort, and an aggressive surgery modality to resect adjacent uninvolved organs has also been proposed by other researchers. Radiotherapy and chemotherapy may further improve prognosis. Generally, this cohort has helped deepen our understanding of LPS and describes the characteristics of a Chinese retroperitoneal LPS cohort.

## 5. Summary

We collected and analyzed the available data from 57 retroperitoneal myxoid liposarcoma patients over an average follow-up time of 4.5 years. Using analysis of current data and comparison with previous studies, we have identified key factors concerning presurgery diagnosis as well as factors influencing prognosis and treatment. The analysis of clinical symptoms, imaging, including CT, ultrasound, and MRI, provides critical evidence when diagnosing LPS and its subtypes. The most important factors deciding prognosis include the LPS subtype, presurgical LPS size, and whether the patient is symptomatic. Among all subtypes, WDLPS had the best prognosis while DLPS and PLPS had the worst. R0 resection is the key treatment for all subtypes, and an aggressive surgery modality to resect uninvolved adjacent organs in DLPS and chemotherapy and radiotherapy for MLPS are also alternative choices.

## Figures and Tables

**Table 1:** Demographic and clinical characteristics of retroperitoneal LPS patients.

Demographic characteristics *n* = 57	
Sex male/female	26/31
Age at diagnosis, mean (SD), years	57.0 (12.2)
Duration of follow-up, mean (SD), years	5.3 (2.6)

Clinical characteristics *n* = 57	
Location	
Retroperitoneum	57 (100%)
Pelvis invasion	7 (12%)
Left retroperitoneum	29 (51%)
Right retroperitoneum	24 (42%)
Both retroperitoneums	4 (7%)

Symptoms	
WDLPS	18
Asymptomatic	10 (56%)
Abdominal distension	6 (33%)
Abdominal pain	2 (11%)
DLPS	23
Abdominal pain	11 (48%)
Asymptomatic	9 (39%)
Abdominal distension	5 (21%)
Lower extremity discomfort	5 (21%)
Fever	2 (9%)
Vomiting	1 (4%)
Frequent micturition	1 (4%)
PLPS	4
Asymptomatic	3 (75%)
Abdominal pain	1 (25%)
MLPS	7
Abdominal distension	4 (57%)
Asymptomatic	3 (43%)
Abdominal pain	2 (29%)
Mixed LPS	6
Abdominal distension	3 (50%)
Asymptomatic	1 (17%)
Lower extremity discomfort	1 (17%)
Dysuria	1 (17%)
Overall	57
Asymptomatic	25 (44%)
Abdominal distension	18 (32%)
Abdominal pain	16 (28%)
Lower extremity discomfort	6 (11%)
Urinary system symptom	2 (4%)

Physical signs	
WDLPS	18
Absent	9 (50%)
Tangible mass	9 (50%)
DLPS	23
Absent	7 (30%)
Tangible mass	16 (70%)
PLPS	4
Tangible mass	4 (100%)
MLPS	7
Absent	2 (29%)
Tangible mass	5 (71%)
Mixed LPS	5
Tangible mass	5 (100%)
Overall	57
Absent	18 (32%)
Tangible mass	39 (68%)

Characteristics of retroperitoneal mass	
WDLPS	9
Clear border	8 (89%)
Good mobility	3 (33%)
Tenderness pain	2 (22%)
DLPS	16
Clear border	9 (56%)
Good mobility	3 (19%)
Tenderness pain	8 (50%)
PLPS	4
Clear border	4 (100%)
Good mobility	1 (25%)
Tenderness pain	1 (25%)
MLPS	5
Clear border	5 (100%)
Good mobility	2 (40%)
Tenderness pain	2 (40%)
Mixed LPS	5
Clear border	3 (60%)
Good mobility	1 (20%)
Tenderness pain	1 (20%)
Overall	39
Clear border	29 (74%)
Good mobility	10 (26%)
Tenderness pain	14 (36%)

Imaging methods	
B ultrasound	9/11 (82%)
CT scan	9/11 (82%)
MRI	2/11 (18%)

MLS: myxoid liposarcoma; CT: computed tomography; MRI: magnetic resonance imaging; CM: centimeter; SD: standard deviation.

**Table 2:** Size of tumor determined by different methods.

No.*∗*	PE	CT	Ultrasound	Resection	Subgroup
1	7*∗*4	12*∗*11	NA	18.5*∗*17*∗*10.3	Small
2	10	NA	11.6*∗*9.6*∗*10.4	15*∗*8*∗*6	Small
3	10*∗*5	10.8*∗*5.4	7.8*∗*5.9	11*∗*7.5*∗*6	Small
4	20*∗*9	17.9*∗*17.7*∗*12.1	15.8*∗*11.7	22.8*∗*19.5*∗*10	Middle
5	Intangible	NA	10*∗*8	20*∗*20*∗*5.5	Small
6	Intangible	9.6*∗*7.6	NA	12.5*∗*8.5*∗*6	Small
7	10*∗*10	17.2*∗*13	No data	18*∗*15*∗*9	Middle
8	Intangible	7.4*∗*6	NA	11.4*∗*9*∗*5.8	Small
9	10	25.2*∗*13.7*∗*10	NA	30*∗*28*∗*17	Large
10	10*∗*10	12.5	14.9*∗*12.9*∗*10.7	9*∗*9*∗*4.8	Small
11	10	12.3*∗*11.0*∗*8.9	NA	16*∗*12*∗*6	Small
12	Intangible	5.7*∗*4.9*∗*3.8	NA	7.5*∗*5*∗*4.2	Small
13	15*∗*15	20.6*∗*16.1*∗*9.7	NA	19*∗*16*∗*6.5	Middle
14	25*∗*20	20*∗*14*∗*25	NA	30*∗*25*∗*10	Middle
15	Intangible	12.9*∗*12.3*∗*17.2	NA	19*∗*15*∗*8	Middle
16	20*∗*15	24.4*∗*17.5*∗*21.4	NA	22*∗*20*∗*4.5	Middle
17	10	21.2*∗*12.9	NA	25*∗*22*∗*9.5	Middle
18*∗*	Intangible	34.3*∗*31.7*∗*23.8	NA	37*∗*30*∗*16.5	Large
19	10	30*∗*30*∗*20	NA	21*∗*18.5*∗*3.5	Large
20	Intangible	4.2*∗*3	NA	5.5*∗*3*∗*1.5	Small
21	Intangible	4.5*∗*4.5*∗*2.7	4.5*∗*3.2	4*∗*3.5*∗*3	Small
22	Intangible	NA	NA	4.5*∗*2*∗*2	Small
23	15*∗*15	15.8*∗*15.3	No data	20*∗*17*∗*12	Middle
24	10	10*∗*9.7*∗*9.6	NA	11*∗*9*∗*8	Small
25	No data	26.5*∗*13.1*∗*36.6	No data	32*∗*40*∗*20	Large
26	25	24.9*∗*23.9*∗*28.2	NA	48*∗*36*∗*12	Middle
27	No data	23*∗*20	25*∗*17	33*∗*21*∗*12	Middle
28	20	NA	25.3*∗*18.8*∗*14.2	15*∗*13*∗*3	Large
29	25*∗*15	16*∗*9.5	NA	25*∗*17*∗*9	Middle
30	18*∗*10	18.0*∗*12.5*∗*11.4	18.6*∗*11.6	18*∗*12*∗*9	Middle
31	No data	30.5*∗*20.9	NA	40*∗*35*∗*20	Large
32	20	NA	17.5*∗*12.2*∗*13.6	30*∗*25*∗*6	Middle
33	30	No data	NA	38*∗*30*∗*8.5	Large
34	Intangible	NA	10.3*∗*5.9	13.5*∗*11.5*∗*8.5	Small
35	12*∗*12	12*∗*12	NA	18*∗*13*∗*7	Small
36	Intangible	8.6*∗*5.5	NA	15*∗*11*∗*5	Small
37	Intangible	8.9*∗*7.3*∗*9.7	NA	11.7*∗*9*∗*9	Small
38	10	14*∗*14*∗*16.5	NA	20*∗*20*∗*12	Middle
39	10*∗*10	14.3*∗*9.7	NA	19.5*∗*15.8*∗*9.5	Middle
40	Intangible	NA	10*∗*7.2	20*∗*16*∗*5	Small
41	25*∗*15	17.6*∗*8.9	NA	15.5*∗*8	Middle
42	12*∗*12	12*∗*8	11.8*∗*9.5*∗*9.2	15*∗*14*∗*14	Small
43	Intangible	12.2*∗*9.9*∗*8.5	NA	15*∗*12*∗*7	Small
44	25	26.4*∗*25.4*∗*16.5	NA	36*∗*32*∗*8	Large
45	Intangible	16*∗*13	20.2*∗*16.1*∗*15.5	23*∗*15.5*∗*14.5	Middle
46	No data	7.5*∗*5.4*∗*6.6	NA	9*∗*8.5*∗*1.5	Small
47	15	19.2*∗*9.3	NA	17*∗*14*∗*8	Middle
48	25*∗*20	NA	NA	15*∗*15*∗*10	Middle
49	Intangible	5.1*∗*4.1	5.6*∗*4.7	13*∗*11*∗*5	Small
50	15*∗*5	6.9*∗*2.5*∗*4.5	13.7*∗*18*∗*6.2	22.5*∗*15*∗*7	Middle
51	Intangible	NA	11.2∗5.3	15*∗*13*∗*4	Small
52	15*∗*10	No data	NA	17*∗*14*∗*12	Small
53	8	13.3*∗*12.0*∗*27.8	15.3*∗*13.2*∗*11.2	25*∗*14*∗*13	Large
54	20*∗*15	24.5*∗*12.7*∗*23.3	14.7*∗*8.48	29*∗*16*∗*11	Middle
55	Intangible	NA	9.3*∗*5.4	15*∗*11*∗*3	Small
56	10*∗*10	9.21*∗*9.95*∗*9.26	NA	16*∗*12*∗*7	Small
57	7*∗*8	16.5*∗*16.2*∗*13.7	11.4*∗*7.4*∗*6.6	16.8*∗*9*∗*6.5	Middle

All data are in centimeters. NA: corresponding examination was not performed. No data: corresponding examination was performed, but no specific number was recorded. ^*∗*^No. 18: although large, the tumor mass was intangible, and the inaccessibility of the tumor may be related to hernia.

**Table 3:** Characteristics of tumor determined by different methods.

Patient no.	PE	Ultrasound-echoic	CT-density	Pathology
1	Tangible	NA	Low	PLPS
2	Tangible	Mixed-echoic	NA	PLPS
3	Tangible	Hypoechoic	Low	PLPS
4	Tangible	Mixed-echoic	Mixed	PLPS
5	Intangible	Mixed-echoic	NA	WDLPS
6	Intangible	NA	Mixed	WDLPS
7	Tangible	Hyperechoic	Low	WDLPS
8	Intangible	NA	Low	WDLPS
9	Tangible	NA	Mixed	WDLPS
10	Tangible	Hyperechoic	Low	WDLPS
11	Tangible	NA	Low	WDLPS
12	Intangible	NA	Mixed	WDLPS
13	Tangible	NA	Mixed	WDLPS
14	Tangible	NA	Low	WDLPS
15	Intangible	NA	Low	WDLPS
16	Tangible	NA	Low	WDLPS
17	Tangible	NA	Low	WDLPS
18	Intangible	NA	Mixed	WDLPS
19	Tangible	NA	Mixed	WDLPS
20	Intangible	NA	Low	WDLPS
21	Intangible	Hypoechoic	Low	WDLPS
22	Intangible	NA	NA	WDLPS
23	Tangible	Mixed-echoic	Low	Mixed LPS
24	Tangible	NA	Mixed	Mixed LPS
25	Tangible	Hypoechoic	Mixed	Mixed LPS
26	Tangible	NA	Mixed	Mixed LPS
27	Tangible	Hypoechoic	Low	Mixed LPS
28	Tangible	Hyperechoic	NA	DLPS
29	Tangible	NA	Low	DLPS
30	Tangible	Hypoechoic	Low	DLPS
31	Tangible	NA	Mixed	DLPS
32	Tangible	Hyperechoic	NA	DLPS
33	Tangible	NA	Mixed	DLPS
34	Intangible	Hypoechoic	Mixed	DLPS
35	Tangible	NA	Mixed	DLPS
36	Intangible	NA	Mixed	DLPS
37	Intangible	NA	Mixed	DLPS
38	Tangible	NA	Low	DLPS
39	Tangible	NA	Low	DLPS
40	Intangible	Hyperechoic	NA	DLPS
41	Tangible	NA	Mixed	DLPS
42	Tangible	Hypoechoic	Low	DLPS
43	Intangible	NA	Low	DLPS
44	Tangible	NA	High	DLPS
45	Intangible	Hypoechoic	Mixed	DLPS
46	Tangible	NA	Mixed	DLPS
47	Tangible	NA	Low	DLPS
48	Tangible	NA	NA	DLPS
49	Intangible	Hypoechoic	Mixed	DLPS
50	Tangible	NA	Low	MLPS
51	Intangible	Hypoechoic	High	MLPS
52	Tangible	Hyperechoic	Low	MLPS
53	Tangible	Hypoechoic	Mixed	MLPS
54	Tangible	Mixed-echoic	Mixed	MLPS
55	Intangible	Hypoechoic	NA	MLPS
56	Tangible	NA	Mixed	MLPS
57	Tangible	Hyperechoic	Mixed	MLPS

NA: corresponding examination was not performed.

**Table 4:** *P* values of DFS and OS of patients with retroperitoneal LPS under different conditions.

*P* value	Pathological	Diameter	Organ invasion	Symptom
DFS (days)	*P*=0.032	*P*=0.009	*P*=0.737	*P*=0.022
OS (days)	*P*=0.930	*P*=0.298	*P*=0.375	*P*=0.466

LPS: liposarcoma; DFS: disease-free survival; OS: overall survival.

## Data Availability

The data used to support the findings of this study are available from the corresponding author upon request.

## Abbreviations

MLS: Myxoid liposarcoma
PE: Physical examination
PUMCH: Peking Union Medical College Hospital
CT: Computed tomography
MRI: Magnetic resonance imaging
FUS: Fused in sarcoma
DNA: Deoxyribonucleic acid
SD: Standard deviation
ICU: Intensive care unit.
